# Functional morphology of giant mole crab larvae: a possible case of defensive enrollment

**DOI:** 10.1186/s40851-016-0052-5

**Published:** 2016-08-26

**Authors:** Nicole R. Rudolf, Carolin Haug, Joachim T. Haug

**Affiliations:** Ludwig-Maximilians-Universität München, Fakultät für Biologie, Biozentrum, Großhaderner Str. 2, 82152 Planegg-Martinsried, Germany

**Keywords:** Giant larva, Zoea, Hippidae, Defensive behavior, Museum material

## Abstract

**Background:**

Mole crabs (Hippidae) are morphologically distinct animals within Meiura, the “short-tailed” crustaceans. More precisely, Hippidae is an ingroup of Anomala, the group which includes squat lobsters, hermit crabs, and numerous “false” crabs. Within Meiura, Anomala is the sister group to Brachyura, which includes all true crabs. Most meiuran crustaceans develop through two specific larval phases. The first, pelagic one is the zoea phase, which is followed by the transitory megalopa phase (only one stage). Zoea larvae are rather small, usually having a total size of only a few millimeters. Zoea larvae of some hippidan species grow significantly larger, up to 15 mm in size, making them the largest known zoea larvae of all anomalan, and probably all meiuran, crustaceans. It has been suggested that such giant larvae may be adapted to a specific defensive strategy; i.e., enrollment. However, to date such giant larvae represent a rarity.

**Methods:**

Eight specimens of large-sized hippidan larvae from museum collections were photographed with a Canon Rebel T3i digital camera under cross-polarized light. Additionally, one of the specimens was documented with a Keyence BZ-9000 fluorescence microscope. The specimen was subsequently dissected to document all appendages in detail. UV light (377 nm) was used for illumination, consistent with the specimen’s autofluorescence capacities. For high-resolution images, composite imaging was applied.

**Results:**

All specimens differ in important aspects from all other known hippidan zoea larvae, and thus probably represent either previously unreported larvae or stages of known species, or larvae of unknown species. The sixth pleon segment articulates off the telson, a condition not previously reported in hippidan zoea larvae, but only for the next larva phase (megalopa). The larvae described here thus most likely represent the ultimate pelagic larval stages, or rare cases of ‘early megalopae’. The morphological features indicate that giant hippidan larvae perform defensive enrollment.

**Conclusions:**

Our investigation indicates a larger morphological diversity of hippidan larvae than was known previously. Moreover, their assumed functional morphology, similar to the condition in certain stomatopod larvae, indicates a not yet directly observable behavior by these larvae, namely defensive enrollment. In a wider context, we are only just beginning to understand the ecological roles of many crustacean larvae.

**Electronic supplementary material:**

The online version of this article (doi:10.1186/s40851-016-0052-5) contains supplementary material, which is available to authorized users.

## Background

Within the diverse group of Eucrustacea, Hippidae is a rather small ingroup with a distinct adult morphology; its representatives are known as sand or mole crabs [1]. Hippidae is an ingroup of Anomala (often also termed Anomura), the group uniting hermit crabs, false crabs, and squat lobsters. Anomala and Brachyura (true crabs) together form Meiura.

Within Hippidae, three species groups are generally differentiated: *Emerita* Scopoli, 1777, *Hippa* Fabricius, 1787 and *Mastigochirus* Miers, 1878 [2]. As with other meiurans, representatives of Hippidae develop through two distinct larval phases: a zoea phase with 3–6 pelagic zoea stages, followed by a critical metamorphic molt into a single megalopa stage representing a still-swimming transitory form [3]. The juvenile and adult stages have a benthic mode of life in intertidal and upper subtidal sandy marine environments [4].

Representatives of Hippidae are special among Meiura in that some of their zoea larvae may achieve impressive sizes. These can reach shield lengths of over 6 mm and, together with the long and slender pleon, may be more than 15 mm long when outstretched [4], whereas most meiuran megalopae are significantly smaller. In fact, these probably represent the largest zoea larvae of all anomalan crustaceans, and possibly all meiurans.

Martin and Ormsby ([4], their Fig. 1b) depicted one such super-sized specimen positioned with a strongly anteriorly flexed pleon. They furthermore pointed out how well the “opercular-like” telson (term from [4]) fits the ventral shape of the shield. While not further discussed in this original work, the function of this tight fit seems most likely to be a specific defensive strategy; more precisely, these larvae appear able to perform defensive enrollment.

**Fig. 1:**
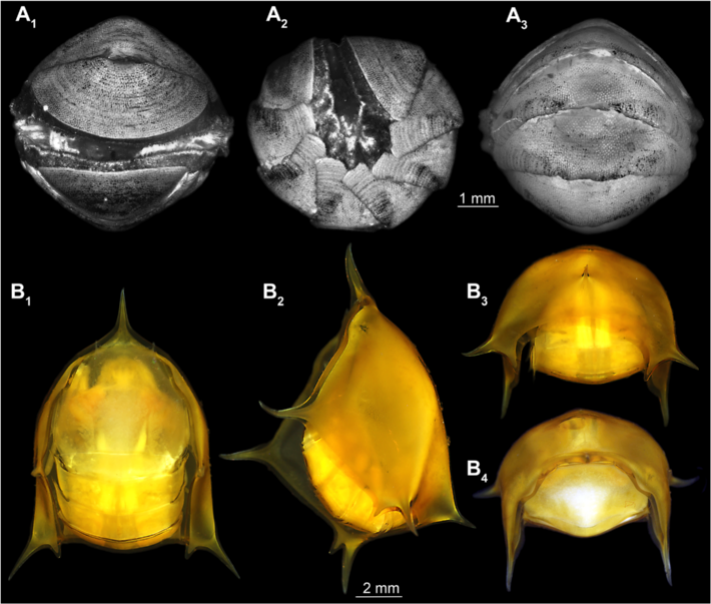
**a**–**b** Commonly known species that exhibit defensive enrollment. **A**_**1**_–**A**_**3**_ Autofluorescence images of *Chiton* spec. (Polyplacophora). **A**_**1**_ Ventral view. **A**_**2**_ Lateral view. **A**_**3**_ Dorsal view. **B**_**1**_–**B**_**4**_ Composite images under cross-polarized light of a mantis shrimp larva (Stomatopoda, Erichthus-type, see [7]). **B**_**1**_ Ventral view. **B**_**2**_ Lateral view. **B**_**3**_ Posterior view. **B**_**4**_ Frontal view

Enrollment is a defensive mechanism which apparently evolved several times independently within Metazoa, often combined with morphological specializations, such as hard plates or large sclerotized spines (e.g., [5–7]). In enrollment, the body is strongly curved ventrally, forming a nearly perfect ball, and the anterior and posterior end lie adjacent to each other. As a result, sclerotized or hardened dorsal structures protect the softer, ventral side of the body and all appendages.

Within vertebrates, armadillos (Xenarthra, Mammalia) are able to bend their body to such an extent that they form a ball (e.g. [8]); their “armor” of dorsal overlapping plates composed of bone with a covering of keratin [9], provides protection in this position. Among mollusks, polyplacophorans (chitons) roll up their bodies to the ventral side when detached from the substrate. In this posture, their dorsal shell plates protect the broad and fleshy foot ([10, 11]; see also Fig. 1a).

In arthropods, enrollment of the body as a protective mechanism against predators and other threats is widespread and primarily known from terrestrial arthropods, e.g., pill bugs and pill millipedes. However, some extinct marine species, e.g., trilobites, also performed enrollment [12–15]. Here again, as described for the other groups, the body is strongly curved ventrally and the tergites (dorsal sclerotisations of the segments) protect the softer ventral side of the body and all the appendages.

More recently it has been reported that certain larval representatives of mantis shrimps (Stomatopoda, Eucrustacea) are also able to tightly enroll their bodies. The pleon is bent forward, constituting a sclerotized protection for the entire body with no major gaps ([7]; see also Fig. 1b).

While the description in Martin and Ormsby [4] indicated the possibility of morphological adaptations for enrollment in hippidan larvae, this appears not to have been investigated further. In the present report, we present new specimens of giant hippidan larvae and provide a detailed description of the general morphology using modern imaging techniques. We discuss morphological details which support the interpretation that these larvae can indeed perform defensive enrollment. We furthermore document an unexpected morphological diversity among giant hippidan larvae.

## Methods

### Materials

Eight hippidan larval specimens were the basis for the present investigation. Six of the specimens came from the zoological collections of the Natural History Museum of Denmark, Copenhagen (ZMUC), registered under the numbers ZMUC-CRU-8679 to 8684. These specimens were collected during the Dana expeditions (1921–22 and 1928–30; Schmidt 1926, 1931; Broch 1936). One specimen came from the crustacean collections of the Senckenberg Naturmuseum Frankfurt (Mu_267), and one from the Muséum national d’Histoire naturelle Paris (MNHN-IU-2014-5468). All are currently stored in 70 % ethanol, probably after previous fixation in formalin. For ventral and dorsal documentation (always within the storage liquid), some specimens were carefully outstretched and fixed with a cover slip. For large specimens, posterior and anterior ends were each fixed with separate cover slips. In other orientations, specimens were either propped against glass or metal objects, or placed into depressions. Specimens in unusual positions were not altered, but kept in this specific position. A single specimen (ZMUC-CRU-8679) was dissected directly in 70 % ethanol using a dissection microscope.

### Documentation

All eight specimens were photographed using a Canon Rebel T3i camera with a MP-E 65 mm macro lens. Light was provided by a Canon Macro Twin Flash MT 24EX or a MeiKe FC 100 LED ring light. Light sources were equipped with polarization filters. A cross-polarized filter was placed in front of the lens. Cross-polarized light reduces reflections and enhances colour contrast (e.g., [16] and references therein). Additionally, one of the eight specimens (ZMUC-CRU-8679) was documented in 70 % ethanol using a Keyence BZ-9000 fluorescence microscope with either a 2×, 4× or 10× objective (resulting in approximately 20×, 40×, and 100× magnification, respectively; in a few cases the zoom function of the camera was also employed) depending on the different sizes of the body parts. UV light (377 nm) was used for illumination, using the autofluorescence capacities of the specimens (e.g. [17]). For high-resolution images, composite imaging was applied [18, 19].

### Image processing

Image stacks were fused with the computer software CombineZP into sharp images. Adobe Photoshop CS3 was used to merge different sharp image details resulting in large panorama images. Finally, images were edited in Adobe Photoshop CS6 (optimization of the histogram and sharpness, manual removing of dirt particles etc. e.g. [7]).

### Drawings

For better comparison, the different telson shapes of the specimens ZMUC-CRU-8679, 8682, and 8683 were drawn in Adobe Illustrator CS 3.

### Presentation

The description is provided as a descriptive matrix (Additional file 1) [20]. This allows a more direct comparison of corresponding structures, which may facilitate future detailed descriptions of other larvae.

### Terminology

Most terms applied are standard crustacean terms (e.g. [21, 22]). However, we have sought to keep terminology neutral to the extent possible, in the interests of allowing comparisons across a wider (arthropod) range. Special terminology of malacostracan or decapod-type is provided in brackets.

## Results

In the following, we describe one of largest specimens (specimen A) in detail. Furthermore, we provide a morphological description of comparable features of the additional seven specimens (specimen B–H). As the latter ones were not dissected, only features that were available in the intact specimens are described.

*Specimen A (ZMUC-CRU-8679):*

**Fig. 2:**
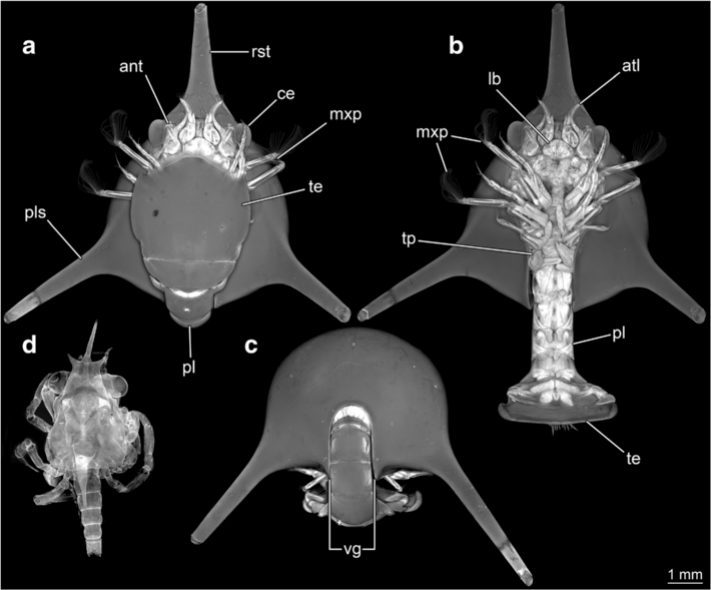
**a**–**d** Autofluorescence images of a hippidan larva (ZMUC-CRU-8679) and a spider crab larva (*Maja* sp.). **a** Ventral view, fully enrolled. **b** Ventral view, fully outstretched. **c** Posterior view. **d** Dorsal view. Abbreviations: ant = antenna; atl = antennula; ce = compound eye; lb = labrum; mxp = maxilliped; pl = pleon; pls = postero-lateral spine; rst = rostral spine; te = telson; tp = thoracopod; vg = ventral gape

*Habitus* (Fig. 2)*.* Small arthropod larva with a globose shield, bearing a long, anteriorly directed, rostral spine (slightly shorter than shield length) and lateral spines (similar length as rostral spine).

*Body* (Fig. 2) differentiated into cephalothorax, pleon and non-somitic telson. Body with 20 segments, ocular segment plus 19 appendage-bearing (post-ocular) segments.

*Ocular segment* incorporated into the cephalothorax, dorsal area contributes to the shield.

*Post-ocular segment 1-13* (Fig. 2) incorporated into the cephalothorax, dorsal area contributes to the shield.

*Post-ocular segment 14-19* (Fig. 2) are separate pleon segments, each dorsally forming a tergite.

*Cephalothorax* (Fig. 2) shield more or less spherical, without setae; large, shield-like, cuticular structure formed by dorsal region of cephalothoracic segments. Anterior rim of the shield drawn out into prominent rostral spine. Posterior rim of the shield slightly convex, with a confined gape, as wide as the posterior gape of the shield. Rostrum unpaired anterior extension of shield, elongated, without spines; anterior region slightly bent upwards. Shield length about 8.5 mm (measured with rostral spine) and 5.1 mm without rostral spine, maximum shield width (measured without spines), about 5.2 mm (about 60 % of shield length with rostral spine). Rostral spine about 40 % of the shield length with rostral spine.

*Post-ocular segment 14* (Fig. 2) anterior-posterior dimension about 25 % of the shield length (without rostral spine); total width of the segment 25 % of the maximum shield width, as wide as the posterior gape of the shield; tergo-pleura not developed; anterior region of post ocular segment 15 slightly convex.

*Post-ocular segment 15* (Fig. 2) anterior-posterior dimension about 5 % of the shield length (without rostral spine). Total width of the segment 25 % of the maximum shield width, as wide as the posterior gape of the shield. Tergo-pleura not developed. Post-ocular segment 15 armed with one cone-shaped spine in the middle of anterior rim of the segment.

*Post-ocular segment 16* (Fig. 2) anterior-posterior dimension about 20 % of the shield length (without rostral spine). Total width of the segment about 25 % of the maximum shield width. Tergo-pleura not developed.

*Post-ocular segment 17* (Fig. 2) anterior-posterior dimension about 20 % of the shield length (without rostral spine). Total width of the segment about 25 % of the maximum shield width. Tergo-pleura not developed.

*Post-ocular segment 18* (Fig. 2) anterior-posterior dimension about 15 % of the shield length. Total width of the segment 50 % of the maximum shield width, measured on posterior rim of the segment. Axial region 25 % of the maximum shield width (without rostral spine). Tergo-pleura about 40 % of the axial region, on each side.

*Post-ocular segment 19* (Fig. 2) anterior-posterior dimension about 10 % of the shield length (without rostral spine). Total width of the segment about 50 % of the maximum shield width. No clear differentiation between axial region and tergo-pleura.

*Telson* (Fig. 2) in dorsal view more or less rectangular. About 45 % of shield length (without rostral spine) and about 30 % wider than long. Anterior rim slightly concave, posterior rim convex. The lateral rim on each side slightly convex, telson width suddenly increased after about 20 % from anterior to posterior rim. Telson shape in lateral view distally tapering. Tip of telson more or less triangular-shaped from dorsal view, with a flattened tip. Forty-seven simple setae on tip of telson. Further lateral setae shorter than distal ones. The 20^th^ setae counted from each terminal rim are the longest ones, the most central one is about 50 % shorter than the longest. Telson armed with two spines on distal rim as protrusion of lateral rim on each side.

*Lateral eyes* (Fig. 2) compound eyes, with numerous ommatidia covered by cornea; stalked.

*Hypostome-labrum complex* (Fig. 2) with more or less triangular-shaped labrum in ventral view, anteriorly surrounded by hypostome.

**Fig. 3:**
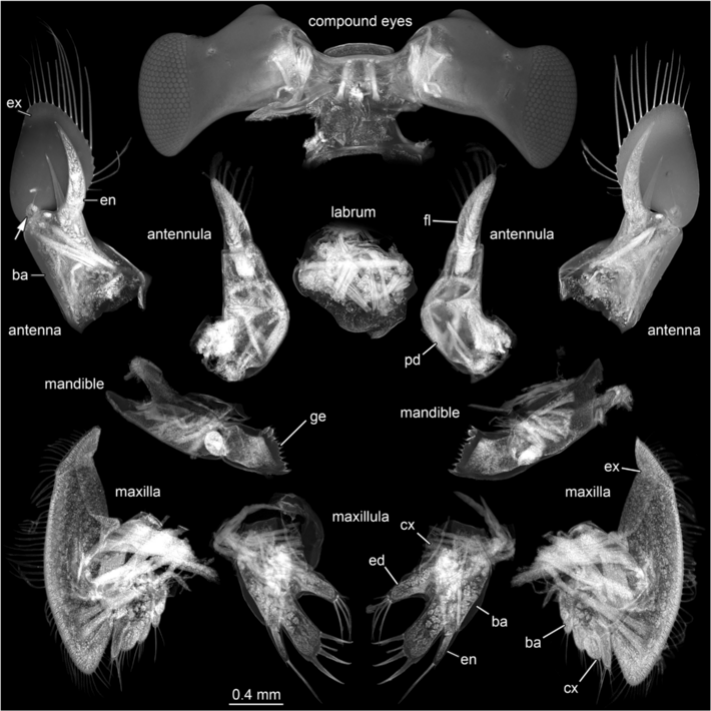
Autofluorescence images of compound eyes, labrum, antennula, antenna, mandible, maxillula, and maxilla of the hippidan specimen (ZMUC-CRU-8679). Abbreviations: ba = basipod; cx = coxa; ed = endit; en = endopod; ex = exopod; fl = flagellum; ge = gnathic edge; pd = peduncle. Arrow: excretory opening

*Appendage 1 (Antennula)* (Fig. 3) differentiated into peduncle and one flagellum. Antennula with aesthetascs. Peduncle more or less tube-shaped and curved to outer lateral rim of the shield. With spine-like protrusion on distal part of the inner lateral rim. Not yet divided into elements, future subdivision into three elements visible. Width of broadest part about 50 % of maximum length. Flagellum 1 not yet developed. Flagellum 2 tube-shaped with a rounded tip. About 35 % shorter than peduncle, with a slightly curved inner lateral rim with numerous setae (aesthetascs) arranged in six tiers.

*Appendage of post-ocular segment 2 (Antenna)* (Fig. 3) differentiated into coxa, basipod (peduncle), endopod and a paddle-shaped exopod; bears opening of antennal gland on basipod. Peduncle not yet divided into elements, with one spine on distal rim of basipod, where endopod arises from it. Endopod pointed and curved, not yet divided into elements, without setae. Exopod paddle-shaped, with 17 plumose setae on the rounded tip and the outer lateral rim.

*Appendage of post-ocular segment 3 (Mandible)* (Fig. 3) differentiated into coxa with endite and mandibular palp. Coxa elongate in medio-lateral axis, medially ending in a row of about 12 teeth. Row consisting of more or less lobate teeth, short triangular and longer elongate teeth with a pointed tip. Mandibular palp not yet developed, but future palp visible. Sternal protrusion of mandibular segment (paragnaths) u-shaped with two lateral elongate paddle-shaped setae bearing protrusions on distal rim. About 35 % wider than maximum length and about as large as hypostome-labrum complex.

*Appendage of post-ocular segment 4 (Maxillula)* (Fig. 3) differentiated into coxa with coxal endite and basipod with basipodal endite and endopod. Coxal endite more or less triangular-shaped from proximal to distal, with a rounded tip, with four elongated plumose setae at the tip. Basipodal endite paddle-shaped, elongate, with four spines at the tip, armed with tiny spines; about 30 % longer than coxal endite. Endopod pointed extension on basipod, not subdivided; one elongate, plumose seta, and one smaller seta on the tip.

*Appendage of post-ocular segment 5 (Maxilla)* (Fig. 3) differentiated into coxa and basipod, both drawn out into two pronounced lobate endites each and exopod. Coxa with two lobate endites with four setae on each lobe. Distal lobe smaller, than proximal one. Basipod with two lobate endites with four setae on each lobe. Distal lobe larger, than proximal one. Endopod not yet developed. Exopod of appendage 5 largest element, bilobed with a distal and proximal lobe; with numerous plumose setae around the rim.

**Fig. 4:**
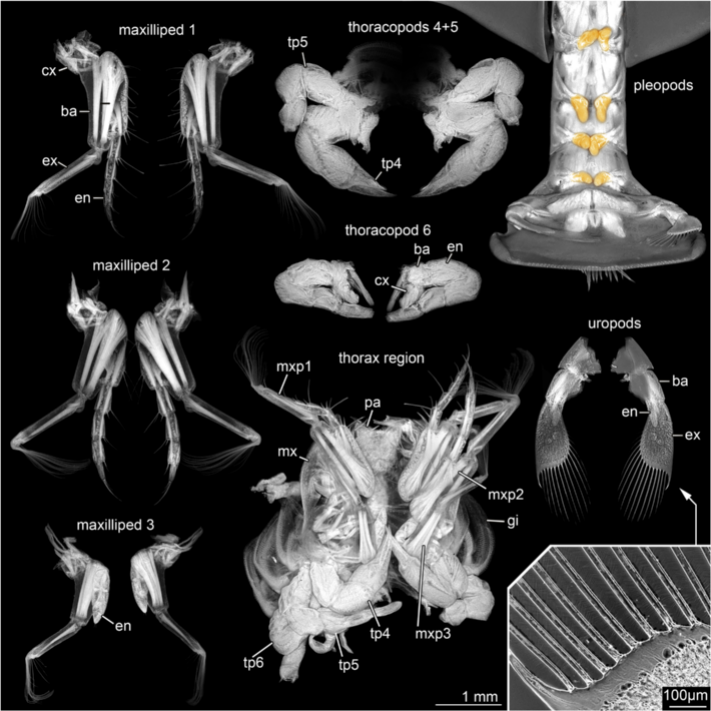
Autofluorescence images of maxillipeds 1–3; thoracopods 4, 5, 6; thorax region; pleopods (orange); uropods (arrow = details of the exopod with setulae-bearing setae). Abbreviations: ba = basipod; cx = coxa; en = endopod; gi = gills; mxp = maxilliped; tp = thoracopod

*Appendage of post-ocular segment 6 (Maxilliped 1)* (Fig. 4) with coxa and basipod, from which endopod and exopod arise. Coxa and basipod with endites. Coxa more or less tube-shaped; with endite. Coxal endite small, more or less triangular from proximal to distal, without setae. Basipod more or less rectangular, with heart-shaped protrusion of anterior part; bears endopod and exopod; about 73 % longer than coxa, about twice as long as wide. Basipodal endite very prominent, slightly curved; with nine plumose setae, and four spines (armed with tiny spines) on inner lateral rim. Endopod with five elements (ischium, merus, carpus, propodus, dactylus); about as long as maximum length of basipod. Endopod element 1 more or less tube-shaped; about 45 % longer than wide, with four plumose setae on distal rim. Endopod element 2 more or less tube-shaped; about as long as preceding element; about 45 % longer than wide, with two plumose setae on distal rim. Endopod element 3 more or less tube-shaped, about 30 % shorter than preceding element; about 40 % longer than wide, with two plumose setae on distal rim. Endopod element 4 more or less tube-shaped; about 20 % shorter than preceding element, about 30 % longer than wide, with three plumose setae on distal rim. Endopod element 5 pointed, about 16 % shorter than preceding element; about 60 % longer than wide, with five plumose setae on distal rim. Exopod tube-shaped, tapering; not yet subdivided into elements; bent backwards. 85 % longer than wide, with about 12 plumose setae on the tip; laterally with one lobate protrusion slightly beyond the inner proximal rim and one lobate protrusion slightly below the tip.

*Appendage of post-ocular segment 7 (Maxilliped 2)* (Fig. 4) with coxa and basipod, from which endopod and exopod arise. Coxa and basipod with endites. Coxa more or less tube-shaped, without setae; coxal endite small, more or less triangular from proximal to distal, without setae. Basipod more or less rectangular-shaped, with heart-shaped protrusion of anterior part; bears endopod and exopod, about 70 % longer than coxa, about twice as long as wide. Basipodal endite very prominent, slightly curved, with 3 plumose setae, on inner lateral rim. Endopod with four elements, about as long as maximum length of basipod. Endopod element 1 more or less tube-shaped, about 40 % longer than wide, with three plumose setae on inner distal rim. Endopod element 2 more or less tube-shaped; about 45 % longer than wide; about 10 % longer than preceding element; with two plumose setae on inner distal rim. Endopod element 3 more or less tube-shaped; about 50 % longer than maximum width, and about the same length than preceding element; with two plumose setae on inner distal rim. Endopod element 4 tapering with a rounded tip; about 60 % longer than wide; about 25 % shorter than preceding element; with three plumose setae on tip. Exopod of appendage 7 tube-shaped, tapering; not yet subdivided into elements; bent backwards, about 10 % shorter than endopod and about 85 % longer than wide; with about 12 plumose setae on the tip; setae bearing tip bent to the inner lateral side; laterally with one lobate protrusion slightly beyond the inner proximal rim and one lobate protrusion slightly below the tip.

*Appendage of post-ocular segment 8 (Maxilliped 3)* (Fig. 4) with coxa and basipod, from which endopod and exopod arise; without endites. Coxa more or less rectangular-shaped, without setae; coxal endite not developed. Basipod more or less tube-shaped; bears endopod and exopod. Endopod about 55 % longer than coxa; about twice as long as wide. Endopod with four elements, separation indicated by faint lines, about 15 % shorter than maximum length of basipod and about 70 % longer than wide. Endopod element 1 more or less tube-shaped, about 20 % longer than maximum width; without setae. Endopod element 2 more or less rectangular shaped; about 30 % wider than maximum length; about 40 % shorter than preceding element; without setae. Endopod element 3 more or less tube-shaped; about 50 % longer than maximum width, and about the same length than preceding element; with two plumose setae on inner distal rim. Endopod element 4 tapering, about as long as maximum width; about 15 % longer than preceding element, with two simple setae on tip. Exopod tube-shaped, tapering. Not yet subdivided into elements; slightly bent backwards; about 15 % longer than endopod and about 80 % longer than wide; with about 18 plumose setae on the tip. Setae bearing tip bent to the inner lateral side. Laterally with one lobate protrusion slightly below the tip.

*Appendage of post-ocular segment 9 (Thoracopod 4)* (Fig. 4) with coxa and basipod (difficult to differentiate in this developmental stage) and endopod; without setae. Endopod of appendage 9 consists of five visible elements (difficult to identify at this early developmental stage) separated by faint lines; distal part of this appendage is modified to a prominent chela. Endopod elements 1–2 probably corresponding to ischium and merus, not yet separated; more or less tube-shaped; about 25 % longer than maximum width. Endopod element 3 (carpus) more or less tube-shaped, curved to the inner side; about 15 % longer than maximum width; about 12 % shorter than preceding element.

Endopod element 4 (propodus) about 45 % longer than maximum width, with outgrowth, which represents the complement of the following element; outgrowth about 35 % shorter than mainpart of the element. Endopod element 5 (dactylus) movable against outgrowth of propodus; tapering, slightly curved; about 50 % of maximum length of preceding element (propodus). Chela is formed by the articulation of element 5 (dactylus) against an outgrowth of element 4 (propodus).

*Appendage of post-ocular segment 10 (Thoracopod 5)* (Fig. 4) with coxa and basipod (difficult to differentiate in this early developmental stage) and endopod. Without setae. Coxa difficult to differentiate in this early developmental stage; without setae. Basipod difficult to differentiate in this early developmental stage; without setae. Endopod consists of five elements (difficult to identify in this early developmental stage); distal part without chela. Endopod element 1 (ischium) more or less triangular-shaped from proximal to distal; about 25 % longer than maximum width; without setae. Endopod element 2 (merus) more or less tube-shaped, slightly curved to inner side; about 40 % longer than maximum length and 40 % longer than preceding element; without setae. Endopod element 3 (carpus) tube-shaped; about 30 % longer than maximum width and about 8 % shorter than preceding element; without setae. Endopod element 4 (propodus) tube-shaped, about as long as maximum width and about 30 % shorter than preceding element; without setae. Endopod element 5 (dactylus) tapering, with a slightly rounded tip; about 80 % longer than maximum width, and about 60 % longer than maximum width; without setae.

*Appendage of post-ocular segment 11 (Thoracopod 6)* (Fig. 4) with coxa and basipod (difficult to differentiate in this early developmental stage) and endopod; without setae.

*Appendage of post-ocular segment 12 (Thoracopod 7)* (Fig. 4) with coxa and basipod and endopod (difficult to differentiate in this early developmental stage, separation indicated by faint lines); without setae.

*Appendage of post-ocular segment 13 (Thoracopod 8)* (Fig. 4) with coxa and basipod and endopod (difficult to differentiate in this early developmental stage, separation indicated by faint lines); without setae.

*Appendage of post-ocular segment 14 (Pleopod 1)* (Fig. 4) not found, not documented.

*Appendage of post-ocular segment 15 (Pleopod 2)* (Fig. 4) differentiated into basipod and endopod. Separation indicated by a faint line. Basipod elongate tube-shaped, about 50 % longer than maximum width. Endopod of appendage 15 tube-shaped, with a rounded tip; about 40 % longer than maximum width, and about 20 % shorter than basipod.

*Appendage of post-ocular segment 16 (Pleopod 3)* (Fig. 4) differentiated into basipod and endopod. Separation indicated by a faint line. Basipod elongate tube-shaped, about 35 % longer than maximum width. Endopod of appendage 16 tube-shaped, with a rounded tip; about 25 % longer than maximum width, and about 35 % shorter than basipod.

*Appendage of post-ocular segment 17 (Pleopod 4)* (Fig. 4) differentiated into basipod and endopod. Separation indicated by a faint line. Basipod elongate tube-shaped, about 35 % longer than maximum width. Endopod of appendage 17 tube-shaped, with a rounded tip; about 40 % longer than maximum width, and about 10 % shorter than basipod.

*Appendage of post-ocular segment 18 (Pleopod 5)* (Fig. 4) differentiated into basipod and endopod. Separation indicated by a faint line. Basipod elongate tube-shaped, about 47 % longer than maximum width. Endopod of appendage 18 tube-shaped, with a rounded tip; about 40 % longer than maximum width, and about 35 % shorter than basipod.

*Appendage of post-ocular segment 19 (Uropod)* (Fig. 4) differentiated into basipod and endopod and exopod. Basipod tube-shaped, 55 % longer than maximum width. Endopod tube-shaped with a rounded tip, about 40 % longer than maximum width. Without setae. Exopod paddle-shaped, with about 16 plumose setae on the distal rim, and the inner lateral rim of appendage. With one spine representing the delineation of setae and extention of the outer rim of exopod.

### Morphological description of comparable features of specimens B–H

**Fig. 5:**
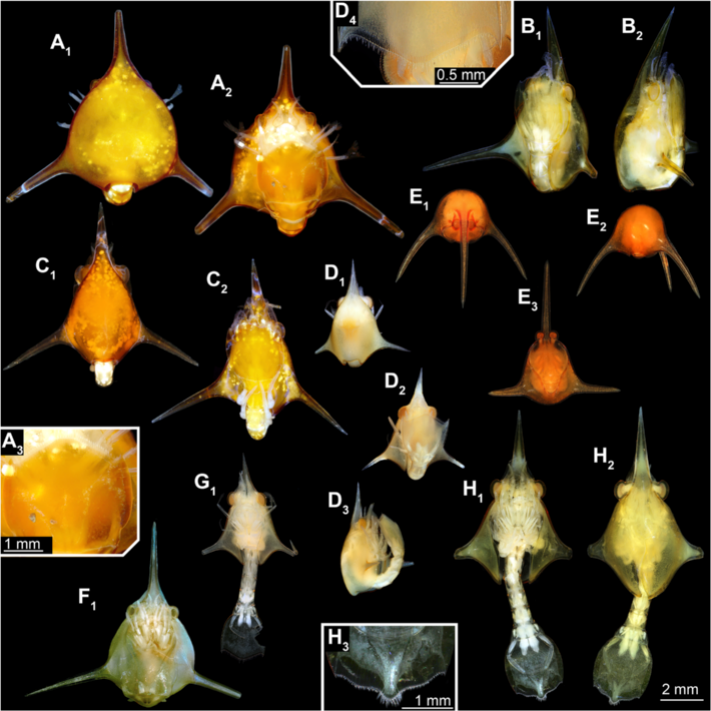
**a**–**h** Composite images under cross-polarized light of hippidan specimens. **A**_**1**_–**A**_**2**_ Dorsal and ventral view (entirely enrolled) of specimen ZMUC-CRU-8679; morphotype 1. **A**_**3**_ Detail of telson of morphotype 1. **B**_**1**_–**B**_**2**_ latero-ventral and lateral view of specimen ZMUC-CRU-8681 (entirely enrolled); morphotype 3. **C**_**1**_–**C**_**2**_ Dorsal and ventral view of specimen ZMUC-CRU-8680 (entirely enrolled); morphotype 3. **D**_**1**_–**D**_**3**_ Dorsal, ventral and lateral view of specimen ZMUC-CRU-8683 (enrolled); morphotype 2. **D**_**4**_ Detail of telson of morphotype 2. **E**_**1**_–**E**_**3**_ Frontal, postero-lateral, and ventral view of specimen MNHN-IU-2014-5468 (entirely enrolled); morphotype 4. **F**_**1**_ Ventral view of specimen Mu_267 (entirely enrolled); morphotype 1. **G**_**1**_ Ventral view of specimen ZMUC-CRU-8684 (entirely outstretched); morphotype 2. **H**_**1**_–**H**_**2**_ Ventral and dorsal view of specimen ZMUC-CRU-8682 (entirely outstretched); morphotype 3. **H**_**3**_ Detail of telson of morphotype 3

*Specimen B (ZMUC-CRU-8681)* (Fig. 5):

Shield. Shield length about 8.5 mm (measured with rostral spine). Maximum width (measured without spines) about 3.5 mm (about 40 % of shield length).

Rostral spine. Anterior part slightly bent upwards. About 45 % of the shield length.

Telson. Anterior and posterior rim slightly convex. The lateral rim on each side slightly convex, width slowly increasing from anterior to posterior. Rim of telson more or less curly brace-shaped, with a rounded tip. Telson armed with two spines on distal rim as protrusion of lateral rim on each side.

*Specimen C (ZMUC-CRU-8680)* (Fig. 5):

Shield. Shield length about 7.5 mm (measured with rostral spine). Maximum width (measured without spines, about 3.4 mm (45 % of shield length).

Rostral spine. Anterior part slightly bent upwards. About 35 % of the shield length.

Telson. Anterior rim slightly convex, posterior rim slightly concave. The lateral rim on each side slightly convex, width slowly increasing from anterior to posterior. Rim of telson more or less curly brace-shaped, with a rounded tip. Telson armed with two spines on distal rim as protrusion of lateral rim on each side.

*Specimen D (ZMUC-CRU-8683)* (Fig. 5):

Shield. Shield length about 5 mm (measured with rostral spine). Maximum width (measured without spines, about 2.3 mm (45 % of shield length).

Rostral spine. Anterior part slightly bent upwards. About 35 % of the shield length.

Telson. Anterior rim slightly convex, posterior rim slightly concave. The lateral rim on each side slightly convex, width slowly increasing from anterior to posterior. Rim of telson more or less triangular-shaped from dorsal view, with a flattened tip. Telson armed with two spines on distal rim as protrusion of lateral rim on each side.

*Specimen E (MNHN-IU-2014-5468)* (Fig. 5):

Shield. Shield length about 7.4 mm (measured with rostral spine) and about 3 mm (measured without rostral spine). Maximum width (measured without spines, about 2.5 mm (30 % of shield length).

Rostral spine. Anterior part strongly bent downwards. About 120 % of the shield length.

Telson. Anterior rim slightly convex, posterior rim slightly concave. Lateral rim difficult to recognize; apparently slightly convex, probably with lobate structure. Telson width suddenly increasing after about 45 % from anterior to posterior rim. Rim of telson more or less triangular-shaped from dorsal view, with a slightly flattened tip. Telson probably armed with two spines on distal rim as protrusion of lateral rim on each side.

*Specimen F (Mu_267)* (Fig. 5):

Shield. Shield length about 8.3 mm (measured with rostral spine). Maximum width (measured without spines), about 4 mm (50 % of shield length).

Rostral spine. No bending visible. Not documented from lateral, or ventro-lateral view. About 50 % of the shield length

Telson. Anterior rim slightly convex, posterior rim slightly concave. Lateral rim slightly convex, with lobate structure. Telson width suddenly increased after about 40 % from anterior to posterior rim. Rim of telson more or less triangular-shaped from dorsal view, with a flattened tip. Telson armed with two spines on distal rim as protrusion of lateral rim on each side.

*Specimen G (ZMUC-CRU-8684)* (Fig. 5):

Shield. Shield length about 5.2 mm (measured with rostral spine). Maximum width (measured without spines), about 2.5 mm (50 % of shield length).

Rostral spine. No bending visible. Not documented from lateral, or ventro-lateral view. About 40 % of the shield length.

Telson. Anterior rim slightly convex, posterior rim slightly concave. The lateral rim on each side slightly convex, width slowly increasing from anterior to posterior. Rim of telson more or less triangular-shaped in dorsal view, with a flattened tip. Telson armed with two spines on distal rim as protrusion of lateral rim on each side.

*Specimen H (ZMUC-CRU-8682)* (Fig. 5):

Shield. Shield length about 9 mm (measured with rostral spine). Maximum width (measured without spines, about 3.6 mm (40 % of shield length).

Rostral spine. Anterior part slightly bent upwards. About 40 % of the shield length.

Telson. Anterior rim slightly convex, posterior rim slightly concave. The lateral rim on each side slightly convex, width slowly increasing from anterior to posterior. Rim of telson more or less curly brace-shaped, with a rounded tip. Telson armed with two spines on distal rim as protrusion of lateral rim on each side.

## Discussion

### Identification of the specimens

**Fig. 6:**
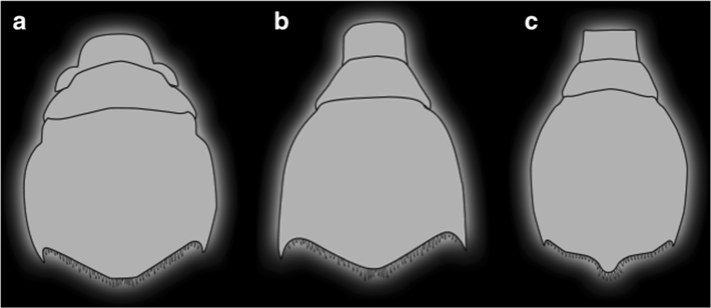
Drawings of the different telson-shapes in dorsal view of the investigated species. **a** Morphotype 1. **b** Morphotype 2. **c** Morphotype 3

The specimens described here show some inter-individual differences, but are sufficiently similar to be discussed together. The overall morphology immediately identifies them as reptantian zoea larvae; the embryonic-like posterior thoracopods identify them as an ingroup of Meiura (cf. e.g. [3, 23]). Most meiuran zoeae possess a forked telson (e.g. [3, 23–25]), while the posterior rim of the telson of the here described specimens has a roughly convex shape in dorsal view (with additional lobate protrusions in some specimens). This shape is known from zoea larvae of hippidan species (Figs. 5, 6). Hippidan larvae, similar to the present specimens, have been described with a more or less spherical shield with a rostral spine and two large spines on each postero-lateral margin, but no postero-dorsal spine. Finally, representatives of Hippidae achieve significantly larger sizes as zoea larvae [3, 4] compared to other meiurans. This is also true for the specimens described here (Figs. 2, 5).

After metamorphosis to the megalopa stage in hippidans, antennula and antenna include a long, setose flagellum, the mandible is divided into two parts, thoracopods are divided into elements and largely resemble the setae-bearing juvenile and adult ones; the pleopods as well as the exopods of the uropods bear setae [4]. The new specimens are therefore interpreted as zoea larvae of species of Hippidae. A late zoea stage is indicated by a differentiation of the non-setae-bearing uropods into an endopod and exopod, by more than three tiers of aesthetascs on the antennula, comparably large maxillipeds, and the presence of primordial thoracopods and pleopods. The details of the appendages of specimen A also strongly resemble known late zoea features (besides their size) [26]. Although the other specimens have not been documented in detail, all are interpreted as representing rather late or final zoea stages.

This is supported in particular by a notable difference to already known zoea larvae of Hippidae: in all specimens the sixth pleomere is already set off from the telson (Figs. 2, 4, 5, 6). Hippidan species for which the larval sequence is known are considered to have the sixth pleomere conjoined to the telson in all zoea stages (forming a pleotelson), becoming finally articulated in the megalopa stage [27].

Hence, in the specimens described here, we probably have ultimate zoea larvae, which could be also interpreted as early megalopae [28], retaining some (in fact most) zoea characters, but already having some megalopa characters. Hippidans are able to vary the number of larval stages (e.g. [29]); these specimens could represent such a case of a prolonged pelagic phase, most likely due to the lack of a settling trigger, which would induce transformation to the megalopa.

### Larvae of mole crabs – what is known so far

Hippoidea includes the groups Blepharipodidae, Albuneidae and Hippidae, the latter two representing sister groups (e.g. [30]). Larval representatives of Albuneidae and Hippidae feature a more or less spherical shield equipped with one prominent lateral spine on each side (absent in larvae of blepharipodids) and one elongate rostral spine [25, 31, 32]. Despite obvious general similarities due to their close relationships, larvae of Albuneidae and Hippidae differ in many aspects [4, 26, 31, 33, 34], assuming the here described specimens are hippidans. A specimen observed by Gurney [25] was referred to as *Albunea* sp. It strongly resembles one of the specimens described herein (Fig. 5h), and is probably also a hippidan larva.

Within Hippidae, there are currently only three species groups recognised: *Emerita*, *Hippa* and *Mastigochirus* (e.g. [4, 26, 33]). Species of *Hippa* and *Emerita* have very similar zoea larvae, with only a few differences. For comparison we refer to the last zoea stage before metamorphosis as the megalopa, since the detailed described larva (specimen A, ZMUC-CRU-8679) is most likely a very late stage zoea.

In larvae of *Hippa* species the rostral spine is slightly curved downwards, but upwards in larval representatives of *Emerita.* Also, the former bear fewer aesthetascs on the antennula, a shorter flagellum on the antenna, fewer setae on the exopod of the maxilla, and fewer setae on the telson. Additionally, larval forms of *Emerita* (as far as known) do not achieve the impressive size of larval forms of *Hippa*. The specimens in the 6^th^ zoeal stage of *Hippa* can reach a shield length of up to 6 mm, whereas zoea larvae in the same stage of *Emerita* only reach a shield length of about 2 mm (see [4, 26, 33]).

### Grouping of material described here

Based on gross morphological aspects, the specimens described here can be sorted into four more or less distinct groups or morphotypes. This will allow an easier comparison with existing descriptions instead of treating each specimen separately.

Morphotype 1 includes specimens A and F. Both share a more spherical shield, a straight rostral spine, a telson with lobate extensions laterally and a flattened tip.

Morphotype 2 includes specimen D and probably G. Both possess a more elongate shield, a straight rostral spine and a telson lacking a lateral protrusion.

Morphotype 3 includes specimens B, C and H. They all possess a shield comparable to morphotype 2, yet the shape of the posterior margin of the telson is not simple and convex (as in morphotype 2), but with a curled, brace-shaped distal margin with a small and rounded lobate tip.

Morphotype 4 is represented by specimen E, which features a more or less spherical shield with a curved rostral spine. The telson of the specimen features lateral protrusions and a flattened tip as in morphotype 1.

### Comparison to the new material

Specimen A (morphotype 1) is quite large, almost reaching the size of a specimen described by Martin and Ormsby [4], which was interpreted as representing the larva of a species of *Hippa*. The rostral spine of the latter specimen is curved downwards, whereas in specimen A the rostral spine is curved slightly upwards.

The antennula of specimen A bears six, rather than the typical five, tiers of setae. It is divided into strongly curved peduncle and flagellum, instead of being not subdivided into a peduncle and flagellum (Fig. 3). The antenna also differs in many aspects. The basipod bears one spine on its distal rim, and there is no flagellum developed as described by Martin and Ormsby [4]. A marked difference constitutes the pointed and strongly curved endopod without setae and the presence of a well-developed paddle-shaped exopod with numerous plumose setae, instead of an endopod with spines and no exopod on the described species of Martin and Ormsby [4] (Fig. 3).

The maxilla has two coxal and two basipodal endites and no endopod, instead of no endites and a setae-bearing endopod. The bilobed exopod and the number of setae of the specimen described here largely resemble the description of Martin and Ormsby [4] (Fig. 3).

The number of telson setae is about twice the number described by Martin and Ormsby [4]. Additionally, the telson is equipped with two lobate structures on each lateral rim (Fig. 4).

Concerning the large size, one might assume that the observed larva is a representative of *Hippa*. However, the considerable morphological differences between the described structures of the specimen of Martin and Ormsby [4] and specimen A (and morphotype 1 in general) does not support its interpretation as a representative of *Hippa*.

Specimen A also differs in many aspects from larvae of species of *Emerita* described by Knight [26]. Concerning the larger number of aesthetascs on the antennula, and the higher number of setae on the exopod of the maxilla and the telson, specimen A resembles larvae of an *Emerita* species. Additionally, the rostral spine of specimen A is bent upwards, as presented in the drawings of Knight [26].

In specimen A, there is no flagellum on the antennula, the antenna bears a paddle-shaped exopod, the telson differs morphologically concerning the lobate structure on the lateral rim, and most strikingly, larvae of *Emerita* are significantly smaller; they achieve a mean size of only about 2 mm shield length [26].

Specimen E (morphotype 4), due to the downwards curvature of the rostral spine and the size of about 3 mm (Fig. 5), matches earlier descriptions of larvae of *Hippa* (e.g. [4]). Hence, specimen E is likely a larval representative of *Hippa*.

The additionally documented specimens, although not investigated in detail, do not show many similarities with the known larvae of *Hippa* or *Emerita* either*.* They also differ among each other (see also below). While based on the size we can hypothesize that the specimens represent late, but different larval stages, not all specimens can be attributed to a single developmental sequence.

Since all specimens described here, except specimen E, differ greatly from earlier described species (Fig. 5), we are unable to determine whether they are larvae of a species of *Emerita*, *Hippa* or *Mastigochirus*. These few specimens indicate that there is still an unknown morphological diversity within larval hippidans. The morphological differences are probably not caused by ontogenetic factors. As discussed above, the larvae most likely either represent ultimate zoea stages or even early megalopa stages. Therefore, they probably do not represent subsequent stages of a single species. The unexpected diversity may indicate that these larvae are individuals of species for which larvae are wholly unknown, differing more significantly than expected from other larval sequences. Yet, it is also possible that they represent special cases of developmental plasticity, which means that they are morphological variations of already known larvae caused by specific environmental conditions. In any case, the morphological diversity of hippidan larvae appears to be higher than that of hippidan adults.

### Enrollment

The specimens described here have not been observed when alive. Still, we can make inferences about their original behavior based on their functional morphology, as recently suggested by Haug and Haug [7]. The basic idea is to employ approaches from paleontology; e.g., recording the preserved position of specimens, identifying specialized morphological features, and comparing these to those of animals with similar features in which behavior can be directly observed. Using these approaches, while any conclusions remain a matter of conjecture, they nonetheless represent an important tool for understanding. So far it has been impossible to breed giant hippidan larvae or to observe them directly in the field. Hence, the approach discussed here is currently the only possibility. Ideally the prediction made here can be tested in the field (see [7] for more details and a comparable example, also corroborated by field observation).

All specimens were originally preserved in an enrolled position, indicating the possibility of the animals to achieve this position. Further morphological adaptations are given for each morphotype separately.

Morphotype 1 (Figs. 2, 5, 6): Specimen A (ZMUC-CRU-8679), and specimen F (Mu_267). In these specimens the shield appears more or less spherical and the posterior gape of the shield has the same width as the pleonal tergites. This combines a spherical shield with maximum mobility of the pleon. The pleon can be stretched out or flexed forward, without any limitations. The large and lid-like telson features a lobate structure on each lateral margin, which ends up in a spine on the distal rim, and the rim of the telson is more or less triangular-shaped in dorsal view and has a slightly flattened tip. The width of the telson and the length of the pleon appear not to be entirely adapted to the ventral gape of the shield. The telson is broader, and due to a comparatively short pleon, the telson does not reach the anterior rim of the shield, even if the pleon is fully flipped forward. Therefore, antennula and antenna as well as the compound eyes and the distal parts of the maxillipeds are not fully concealed.

Morphotype 2 and morphotype 3 (Fig. 5, 6): Specimen D (ZMUC-CRU-8683) and probably specimen G (ZMUC-CRU-8684); respectively specimen B (ZMUC-CRU-8681), specimen C (ZMUC-CRU-8680) and specimen H (ZMUC-CRU-8682). The two morphotypes differ from each other only in the shape of the posterior rim of the telson, but otherwise are quite similar and therefore treated together.

Similar to morphotype 1, both morphotypes feature a posterior gape which has the same width as the pleon. In both morphotypes, the width of the telson is not adapted to the ventral gape of the shield. The telson is wider than the ventral gape and has a different shape. Yet, when flipped forward, the ventral gape is entirely covered across its width.

The large telson has slightly convex lateral margins, which end up in a spine on each side, and the telson width increases slightly from anterior to posterior. Additionally, due to a longer pleon the larvae are able to flex the telson far anteriorly so that the ventral gape of the shield is entirely closed and almost all parts of the appendages are covered by the telson, which perfectly protects the entire body. Only the compound eyes and the distal parts of antennula and antenna remain exposed.

Morphotype 4 (Fig. 5, 6): Specimen E (MNHN-IU-2014-5468) mainly resembles morphotype 1 in relation to the more or less spherical shield shape, the ventral gape and the shape of the telson. As in morphotypes 2 and 3, the pleon is flexed far anteriorly. Additionally, the telson is positioned inside the shield and reaches the anterior rim (Fig. 5). All appendages, except the eyes and the distal tip of the antennulae are protected by the shield, pleon and telson. The fully enrolled specimen has the appearance of a compact ball, armed with spines.

Here the position of the telson is most important. The telson appears to perfectly fit into the shield, with “rail-like” protrusions of the shield keeping it in place. In this position, the telson is tightly locked in place, and the dorsal area of the pleon perfectly closes the posterior gape. With this, the lateral rim of the telson and the ventral rim of the shield apparently form coaptative structures that tightly enclose the enrolled body. The animal probably achieves this position by 1) flexing its pleon forward, 2) pressing it towards its ventral side right in front of the mouthparts and maxillipeds, and 3) sliding it back. During the last step, the lid-like telson is pulled inside the ventrally curved rims of the shield. As a result, the animal secures the enrolled position and achieves full protection of the ventral appendages. Interestingly, this mechanism appears to be arranged “the-other-way-round” as compared to the coaptative structures in stomatopod larvae where the telson is pushed forward in order to lock it [7].

### So do giant hippidan larvae perform defensive enrollment?

Based on our observations we can state that:

1. all specimens are preserved in an enrolled position, indicating that the animals can achieve this position;
2. the shield is large and drawn out ventrally for some distance, unlike in many other decapod zoea larvae, and it is able to house most of the appendages;
3. the width of the shield and the width of the pleon are perfectly adapted to one another; this is not a widespread phenomenon (see Discussion in [7]) and can hence be interpreted as an adaptation for performing enrollment;
4. a large lid-like telson covers most of the ventral gape if flipped forward; also this morphology is rather unusual for meiurans (where the pleon is usually forked); thus it is also possible that this morphology represents a further adaptation for enrollment;
5. at least for morphotype 4 (specimen E) we have indications that there are coaptative structures; such structures are strong indicators of defensive enrollment, as these are developed in other groups for which enrollment seems now corroborated, such as trilobites ([15], their figs. 1E–F; [35, 36]) or stomatopod larvae ([7]).

Hence, for morphotype 4 there should be little doubt about whether it was able to perform enrollment. For the other three, the stronger argument of the coaptative structures cannot be used. Yet, as in the discussion about different morphotypes of stomatopod larvae [7], the presence of only some of the adaptations cannot be used as an argument to exclude this behavior. Also among other animals which are known to perform defensive enrollment coaptative structures appear to be absent (e.g. polyplacophorans; stonefly larvae of *Pteronarcys dorsata* [37]).

With this, we consider it likely that all specimens were able to perform defensive enrollment, but to differing degrees of specialization. The ventral “softer” part of the body is in all cases concealed by the spine-bearing shield and the sclerotized pleonal tergites. Yet, in morphotype 1 (specimens A, F) there is a larger unconcealed region remaining up to the anterior margin of the ventral gape of the shield (Fig. 5; similar to a stomatopod larva morphotype, [7]). Morphotypes 2 (specimens D, G) and 3 (specimens B, C, H) achieve the same defensive effectiveness since they flex the telson further anteriorly, and the ventral gape of the shield is almost entirely closed (Fig. 5). Morphotype 4 (specimen E) achieves the most effective degree of defense since there is no unconcealed region left up to the anterior margin of the ventral gape, and due to the position of the telson inside the shield (Fig. 5).

### Comparison to Brachyura and Stomatopoda

Martin and Ormsby [4] have stated that hippidan larvae appear very similar to brachyuran larvae. This seems to be largely attributable to the shield morphology. Brachyuran zoeae also feature a spherical shield; yet here the rostral spine is directed more ventrally (e.g. [38]) instead of being anteriorly directed as in most hippidan larvae (Fig. 5). Additionally, brachyuran zoea larvae have no uropods [39, 40], whereas hippidan larvae feature uropods from their early zoeal stages onwards [26] (Figs. 2, 4, 5). Brachyuran zoea larvae have two lateral spines and the rostral spine (as hippidan zoeae), but additionally a long postero-dorsal spine, which is absent in hippidan larvae (Figs. 2, 5). Also the telson differs morphologically. There is a pronounced furca with a medial cleft in brachyurans (at least in early zoea stages; [23, 34]. Hippidan larvae never have a forked telson (Figs. 2, 3, 4 and 5), but the posterior rim is convex.

Most strikingly, brachyuran larvae do not achieve the giant size of hippidan larvae; brachyuran larvae with small body lengths seem to be very common (e. g. [41, 42]). Discrete lengths are rarely stated for brachyuran larvae. A shield length (without the rostral spine) of 0.88 mm in an advanced zoeal stage has been reported [34]. The largest late stage zoea in our investigation (specimen F, Mu_267) reaches a shield length of about 5.5 mm (Fig. 5); 6 mm has been reported [4].

There have been no reports to date of defensive enrollment in brachyuran zoea larvae, nor do we see morphological adaptations for it, but that may change if such features are searched for directly. Currently, only a roughly spherical shield with three spines seems to be similar between brachyuran and hippidan zoeae (Fig. 2).

Stomatopods (mantis shrimps) are also discussed here in light of their similarities to hippidan zoea larvae, since some mantis shrimp larvae can also tightly enroll their bodies [7] (Fig. 1). Interestingly, the specimens described here were found between mantis shrimp larvae in two museum collections (see Methods for details; also in other collections, pers. obs.) in roughly pre-sorted samples. This shows nicely how similar mantis shrimp and hippidan larvae appear at first sight.

Also here especially the shield appears similar, even more similar than to brachyuran larvae as stomatopod larvae, like hippidan zoea larvae, lack the pronounced postero-dorsal spine of brachyuran zoeae (Figs. 1, 5). A future, more intensive functional comparison of hippidan and stomatopod larvae could reveal the evolutionary mechanisms leading to the morphological adaptation coupled to defensive enrollment.

## Conclusions and Prospects

Our investigation indicates a broader morphological diversity of hippidan larvae than has been described previously. The functional morphological aspects of these larvae suggest a behavior by these larvae that has not been directly observable to date. It thus appears that we are just starting to understand the ecological roles played by many crustacean larvae. Hence, we expect to continue to uncover hidden morphological diversity among these larvae, and will seek to reconstruct their functional morphology and evolutionary history.

## Additional file

Additional file 1: Descriptive matrix of specimen A (ZMUC-CRU-8679). (XLS 39 kb) Additional file 2: Persistent identifiers for specimens in database MorphDBase. (XLS 7.50 kb)

## Abbreviations

ANT: Antenna
ATL: Antennula
BA: Basipod
CE: Compound eye
CX: Coxa
ED: Endit
EN: Endopod
EX: Exopod
FL: Flagellum
GE: Gnathic edge
GI: Gills
LB: Labrum
MXP: Maxilliped
PD: Peduncle
PL: Pleon
PLS: Postero-lateral spine
RST: Rostral spine
TE: Telson
TP: Thoracopod
VG: Ventral gape
